# Impact of Bedside Ultrasound on Emergency Department Length of Stay and Admission in Patients With a Suspected Peritonsillar Abscess

**DOI:** 10.7759/cureus.32207

**Published:** 2022-12-05

**Authors:** Christopher Bryczkowski, William Haussner, Mary Rometti, Grant Wei, Daniel Morrison, Rajesh Geria, Jonathan V Mccoy

**Affiliations:** 1 Emergency Medicine, Rutgers Robert Wood Johnson Medical School, New Brunswick, USA

**Keywords:** emergency medicine ultrasound, pta, ed length of stay, pocus, peritonsillar abscess

## Abstract

Introduction

Patients presenting to the Emergency Department (ED) with a suspected peritonsillar abscess (PTA) often pose a diagnostic dilemma, as clinical impression is often unreliable and traditional diagnostic methods have multiple downsides. Bedside ultrasonography has been cited as a modality to improve the diagnosis and management of PTA. We aimed to determine the impact bedside ultrasound (US) could have in suspected PTA on ED length of stay (LOS) and hospital admission rates.

Methods

We performed a retrospective chart review on patients who presented to the ED with suspected ‘‘peritonsillar abscess’’.

Results

From a sample of 58 charts, seven had documented bedside US performed. The average ED length of stay for these seven cases was 160 minutes (range: 52 to 270 minutes). The ED length of stay for all other cases utilizing other diagnostic methods during the same time period was 293 minutes (range: 34 to 780 minutes). None of the patients who were diagnosed with US were admitted to the hospital, whereas 36.4% of patients where US was not used were admitted.

Conclusion

The use of bedside US in seven cases of suspected PTA had reduced LOS in the ED and none required hospital admission.

## Introduction

Peritonsillar abscess (PTA) has been cited as the most common deep infection of the head and neck [1-8], with an incidence of one in 10,000 in adolescents and young adults [7,9-11]. The literature has also noted an increase in the incidence of PTA presentation, with one study finding an increase of approximately 18% over a 10-year period [12]. Anatomically, the area of infection forms in the potential space between the palatine tonsils and capsule formed by the intrapharyngeal aponeurosis [11]. Presentation of PTA classically involves odynophagia, a ‘hot potato’ voice, trismus, erythema, edema, and contralateral soft palate deviation [1,13]. Diagnosing PTA on clinical signs alone can be erroneous, as the classic presentation mimics other infections, such as peritonsillar cellulitis [4,6-8,14]. Using only a physical exam to diagnose a PTA has a sensitivity of 78% and a specificity of 50% [15]. Similar to PTA, peritonsillar cellulitis also presents with erythema and bulging of the peritonsillar area [14]. The two diseases are often indistinguishable, so diagnosis is made via needle aspiration. However, needle aspiration has been reported to have a sensitivity of 75%, specificity of 50%, and a false negative rate of 10-24% [4,6,9,14,16]. The treatment of peritonsillar cellulitis and PTA differs, with the latter requiring drainage in contrast to antibiotic therapy for peritonsillar cellulitis.

As a result, performing a needle aspiration to guide the diagnosis may have potential life-threatening consequences. Incomplete treatment of PTA can have severe clinical implications; the infection can spread into neighboring tissues such as the pharynx and trachea, potentially compromising the airway. Spread into the neck can result in carotid artery dissection via vascular erosion, mediastinal infiltration can lead to mediastinitis and cardiac tamponade, and hematogenous seeding can result in sepsis [1,4,7,14,15,17].

The diagnostic inaccuracy of current practices has demonstrated a need for a more accurate diagnos­tic method for PTA. Swift diagnosis and treatment of patients with PTA is crucial; however, ruling out the diagnosis in favor of peritonsillar cellulitis can reduce patient morbidity as well. Patients misdiagnosed with PTA may be subject to unnecessary drainage attempts, which are both invasive and painful [7,15]. If performed blind (without the aid of imaging to guide needle insertion during aspiration) there is a false negative rate of 10-24%, and risk of carotid artery trauma, false aneurism formation, or carotid puncture [1,2,9,16]. Consequently, emergency clinicians may be inclined to utilize Ear, Nose, and Throat (ENT) consultation or computed tomography (CT) of the neck as traditional methods rather than diagnose and treat bedside [11]. However, without the utilization of ultrasound (US) in their procedures, consults will face the same issues of diagnosis and treatment as experienced by emergency clinicians. Such practice is not only unnecessary for the majority of cases, but also wastes resources, time, and has little to no benefit to the patient.

Utilization of diagnostic imaging has been cited as a method to aid in the proper diagnosis of PTA. CT improves upon prior diagnostic methods with a sensitivity of 100% and specificity of 75% and with its ability to delineate if an infection is localized to the tonsillar area has often become the preferred method of diagnosis [4,7,9]. Even so, it is hindered by its cost, wait time, and radiation exposure [4,9,13,15]. US possesses somewhat similar diagnostic accuracy to CT with sensitivity and specificity ranging from 89-95% and 79-100%, respectively [1,5-7,10,14-16,18]. A small study published in 2012 concluded intraoral US can differentiate peritonsillar abscess from peritonsillar cellulitis; furthermore, 64% of attempted needle aspiration drainage by landmarks only were unsuccessful, compared to 0% unsuccessful drainage attempts using intraoral ultrasound [11]. US also has the added advantage over CT via its cost, portability, speed, and lack of radiation [10,15]. Additionally, if the diagnosis of PTA is made successfully, bedside US offers the added benefit of procedural guidance during the aspiration [5,15]. This approach can maximize needle accuracy, promote proper drainage of the site, determine if the infection is localized, and decrease inadvertent carotid artery puncture [1,15]. A study published in otolaryngologist literature found that while US-guided drainage was not tolerated by all patients in their study (due to trismus, tongue movements, or dental issues), when it was tolerated, US had 100% sensitivity [18].

Despite all of the aforementioned benefits, US for PTA is currently underutilized in many emergency departments [12]. There has been no consensus in the management of PTA in the Emergency Medicine (EM) community. Thus, we attempted to determine if intraoral US by emergency clinicians is efficacious in the diagnosis of PTA. We analyzed seven cases in which US was effectively utilized by the emergency physician in order to diagnose and treat PTA.

This article was previously presented at the American Institute of Ultrasound in Medicine Annual Meeting 2015.

## Materials and methods

This was a retrospective case series that focused on the use of bedside US in patients with suspected PTA. A retrospective chart review of all patients over 21 years of age, who presented to the ED at an academic level one trauma center, between January 2011 to February 2014 with the billing code “peritonsillar abscess” was performed. Patients that met these inclusion criteria were given a subject identifier from the beginning of chart analysis and all patient-identifying demographics were removed. Exclusion criteria were patients less than 21 years of age, lack of suspected peritonsillar abscess, or falling outside of the study date range. All ultrasound diagnostic scans and/or procedures are recorded and stored in a quality control database “Qpath” (Telexy Healthcare, Maple Ridge, BC, Canada). All bedside US was performed using endocavitary probes using ultrasounds from the following manufacturers: Zonare ZS3, Zonare Z One Ultra (Zonare Medical Systems, Inc., Mountain View, CA), and Sonosite M-Turbo (Fujifilm Sonosite Inc., Seattle, WA).

The ED arrival time and discharge times were obtained from electronic medical records and length of stay was determined from these data points. Ultimate patient dispositions (admit vs discharge) were noted. In patients that had bedside ultrasonography performed, the use of CT scan, ENT consultations, and presence of any return visits within 30 days were recorded. Any alternate diagnoses after suspected PTA were also recorded.

The statistical analysis of this project utilized t-tests to present the mean emergency department length of stay as it had normally distributed data. If data was not normally distributed, the appropriate non-parametric would have been utilized; however, this was not necessary.

## Results

From a sample of 58 charts of suspected PTA, 51 patients were managed via traditional methods, and seven had documented bedside ultrasonography performed. Of the seven patients who received intraoral US, four patients were diagnosed with a peritonsillar abscess. These patients had US-guided abscess drainage performed by an emergency clinician. From these cases, three patients received intravenous (IV) antibiotics in the ED and two patients were given a steroid injection. All were discharged on oral antibiotics. Additionally, one patient was diagnosed with pharyngitis, one with tonsillitis, and another with peritonsillar cellulitis after the US did not reveal a peritonsillar collection. The patient diagnosed with pharyngitis was seen two days prior and diagnosed with the same. The remaining patients had no prior visits. CT scans and ENT consultations were not obtained in any US patient.

The ED length of stay for all other cases of suspected PTA during the same time frame was 293 minutes (range: 34 to 780 minutes) (Table 1). The average ED length of stay for the seven bedside ultrasound cases was 160 minutes (range: 52 to 270 minutes) (Table 2). None of the bedside ultrasound patients were admitted, and there were no return visits within the subsequent 30 days. Of the patients who did not receive ultrasound, 36.3% of these patients were admitted (Table 3).

**Table 1:** Length of stay and disposition of patients with suspected PTA who did not receive bedside US. PTA: Peritonsillar abscess; US: Ultrasound.

Study ID	Length of Stay (min)	Disposition
1	237	admitted
2	345	admitted
3	458	admitted
4	357	home
5	156	home
6	106	admitted
7	780	home
8	261	home
9	200	home
10	280	admitted
11	87	home
12	439	admitted
13	400	home
14	259	home
15	647	home
16	230	admitted
17	490	home
18	136	home
19	516	home
20	116	home
21	235	home
22	258	home
23	300	admitted
24	409	home
25	185	home
26	319	home
27	231	admitted
28	202	home
29	234	home
30	333	admitted
31	337	home
32	103	home
33	132	admitted
34	58	home
35	173	admitted
36	144	admitted
37	581	home
38	302	admitted
39	288	admitted
40	339	admitted
41	493	home
42	299	home
43	486	home
44	237	home
45	458	admitted
46	357	home
47	156	admitted
48	106	home
49	170	home
50	780	admitted
51	261	home

**Table 2:** Length of stay, disposition, and diagnosis of patients with suspected PTA who did receive bedside US. PTA: Peritonsillar abscess; US: Ultrasound

Study ID	Length of Stay (min)	Disposition	Diagnosis
1	229	home	pharyngitis
2	173	home	PTA
3	270	home	PTA
5	192	home	PTA
4	132	home	tonsillitis
6	52	home	PTA
7	72	home	cellulitis

**Table 3:** Comparison of length of stay and admission rate of patients with suspected PTA diagnosed via US or traditional methods. T-Test p=0.0300 PTA: Peritonsillar abscess; US: Ultrasound

	Average Length of Stay (min)	Admission Rate
Bedside Ultrasound Performed	160 +/- 79.84	0%
No Ultrasound	303.3 +/- 165.10	36.30%

## Discussion

Peritonsillar abscess is a common and potentially life-threatening infection faced in the emergency department. This study focused on the value of ultrasound as a tool for emergency clinicians confronting suspected PTA. The use of bedside ultrasonography in seven cases of suspected peritonsillar abscess was associated with an ED length of stay of 160 minutes. This length of stay (LOS) is shorter when compared to 293 minutes for cases where bedside ultrasonography was not performed. Furthermore, out of the seven cases analyzed in this study, three had an alternate diagnosis after bedside US which were not consistent with PTA. These alternate diagnoses included known mimics of PTA such as pharyngitis, tonsillitis, and peritonsillar cellulitis. Lastly, it was determined that all patients who were diagnosed and managed via US were discharged, whereas 36.4% of patients who did not receive bedside ultrasound and drainage were admitted.

If used routinely in the ED for suspected PTA, this study demonstrates the potential US could have in reducing ED length of stay and the reduction in hospital admissions. A 2012 randomized control trial of US versus needle aspiration supports our data; they found that US was reliable in diagnosis and was superior to diagnosis via needle aspiration or physical exam findings alone [11,17].

Further literature on this topic is sparse. Our study found no return visits in the US group and a marked reduction in length of stay compared to the traditional diagnostic group. Similar results were found in a study by Gibbons and Costantino, where 4.5% of ultrasound patients returned within one week versus 12% of the no ultrasound group [4]. The decreased length of stay that was found in the US group was speculated to be due to the cumulative effects of US: achieving a quicker time to diagnosis and abscess drainage.

In addition, from the seven cases in our study, three were identified to have diagnoses other than PTA. The use of US spared patients the time, expense, and discomfort of unnecessary aspirations. We hypothesize that a shorter LOS would also be reflected in cases in which PTA was excluded utilizing bedside US.

The results of this observational study are limited by multiple factors. A small sample size can result in possible bias in the results. Also, ED length of stay is multifactorial and is often affected by factors other than the one studied in this patient cohort. ED patient volume, nursing and consultant availability among other elements can play a big role. In addition, return visits for those treated without ultrasound (CT and/or consultant drainage) were not recorded. Finally, emergency clinicians’ familiarity with US-guided PTA drainage could also have affected if they elected to perform this procedure. Further research on this topic which would aim to measure the number of tonsillar punctures and adverse events is still necessary.

The results that we have obtained emphasize the continued evidence supporting bedside ultrasound effectiveness in PTA diagnosis and drainage, as well as the reduction in LOS and hospital admissions. Increased use of this diagnostic and therapeutic modality by emergency clinicians could lead to improved diagnostic accuracy and patient satisfaction with decreased dependence on alternate imaging which uses radiation.

## Conclusions

Bedside ultrasound is a valuable tool in the diagnosis and management of suspected peritonsillar abscesses. In this study, the use of bedside ultrasonography in seven cases of suspected peritonsillar abscesses was associated with an ED length of stay that was shorter when compared to cases in which bedside ultrasonography was not performed. None of the patients undergoing US-guided drainage required admission. Further research investigating this treatment modality is warranted.

## References

[1] Lyon M, Blavias M (2005). Intraoral ultrasound in the diagnosis and treatment of suspected peritonsillar abscess in the emergency department. Acad Emerg Med.

[2] Menegas S, Moayedi S, Torres M (2021). Abscess management: an evidence-based review for emergency medicine clinicians. J Emerg Med.

[3] Ortega BK, Short S, Kane BG, Dedio R (2021). Management of peritonsillar abscess within a local emergency department: a quality analysis study. Cereus.

[4] Gibbons RC, Costantino TG (2020). Evidence-based medicine improves the emergent management of peritonsillar abscesses using point-of-care ultrasound. J Emerg Med.

[5] Kolikof JS, Hoffmann B, Schafer JM (2022). A low-cost ultrasound phantom for peritonsillar abscess drainage training. J Emerg Med.

[6] Ng V, Plitt J, Biffar D (2018). Development of a novel ultrasound-guided peritonsillar abscess model for simulation training. West J Emerg Med.

[7] Secko M, Sivitz A (2015). Think ultrasound first for peritonsillar swelling. Am J Emerg Med.

[8] (2022). Ultrasound G.E.L. - POCUS for peritonsillar abscess. http://www.emdocs.net/ultrasound-g-e-l-pocus-for-peritonsillar-abscess.

[9] Scott PM, Loftus WK, Kew J, Ahuja A, Yue V, van Hasselt CA (1999). Diagnosis of peritonsillar infections: a prospective study of ultrasound, computerized tomography and clinical diagnosis. J Laryngol Otol.

[10] Araujo Filho BC, Sakae FA, Sennes LU, Imamura R, de Menezes MR (2006). Intraoral and transcutaneous cervical ultrasound in the differential diagnosis of peritonsillar cellulitis and abscesses. Braz J Otorhinolaryngol.

[11] Costantino TG, Satz WA, Dehnkamp W, Goett H (2012). Randomized trial comparing intraoral ultrasound to landmark-based needle aspiration in patients with suspected peritonsillar abscess. Acad Emerg Med.

[12] Powell J, Wilson JA (2012). An evidence-based review of peritonsillar abscess. Clin Otolaryngol.

[13] Todsen T, Stage MG, Holst Hahn C (2018). A novel technique for intraoral ultrasound-guided aspiration of peritonsillar abscess. Diagnostics (Basel).

[14] Blaivas M, Theodoro D, Duggal S (2003). Ultrasound-guided drainage of peritonsillar abscess by the emergency physician. Am J Emerg Med.

[15] Froehlich MH, Huang Z, Reilly BK (2017). Utilization of ultrasound for diagnostic evaluation and management of peritonsillar abscesses. Curr Opin Otolaryngol Head Neck Surg.

[16] Rehrer M, Mantuani D, Nagdev A (2013). Identification of peritonsillar abscess by transcutaneous cervical ultrasound. Am J Emerg Med.

[17] Fordham MT, Rock AN, Bandarkar A (2015). Transcervical ultrasonography in the diagnosis of pediatric peritonsillar abscess. Laryngoscope.

[18] Nogan S, Jandali D, Cipolla M, DeSilva B (2015). The use of ultrasound imaging in evaluation of peritonsillar infections. Laryngoscope.

